# Protocol for evaluating the effects of a therapeutic foot exercise program on injury incidence, foot functionality and biomechanics in long-distance runners: a randomized controlled trial

**DOI:** 10.1186/s12891-016-1016-9

**Published:** 2016-04-14

**Authors:** Alessandra B. Matias, Ulisses T. Taddei, Marcos Duarte, Isabel C. N. Sacco

**Affiliations:** Department of Physical Therapy, Speech, and Occupational Therapy, School of Medicine, University of São Paulo, Rua Cipotânea, 51 - Cidade Universitária, 05360-160 São Paulo, São Paulo Brazil; Federal University of ABC, Biomedical Engineering, São Bernardo, São Paulo Brazil

**Keywords:** Running, Sports injuries, Exercise therapy, Foot, Biomechanics

## Abstract

**Background:**

Overall performance, particularly in a very popular sports activity such as running, is typically influenced by the status of the musculoskeletal system and the level of training and conditioning of the biological structures. Any change in the musculoskeletal system’s biomechanics, especially in the feet and ankles, will strongly influence the biomechanics of runners, possibly predisposing them to injuries. A thorough understanding of the effects of a therapeutic approach focused on feet biomechanics, on strength and functionality of lower limb muscles will contribute to the adoption of more effective therapeutic and preventive strategies for runners.

**Methods/Design:**

A randomized, prospective controlled and parallel trial with blind assessment is designed to study the effects of a "ground-up" therapeutic approach focused on the foot-ankle complex as it relates to the incidence of running-related injuries in the lower limbs. One hundred and eleven (111) healthy long-distance runners will be randomly assigned to either a control (CG) or intervention (IG) group. IG runners will participate in a therapeutic exercise protocol for the foot-ankle for 8 weeks, with 1 directly supervised session and 3 remotely supervised sessions per week. After the 8-week period, IG runners will keep exercising for the remaining 10 months of the study, supervised only by web-enabled software three times a week. At baseline, 2 months, 4 months and 12 months, all runners will be assessed for running-related injuries (primary outcome), time for the occurrence of the first injury, foot health and functionality, muscle trophism, intrinsic foot muscle strength, dynamic foot arch strain and lower-limb biomechanics during walking and running (secondary outcomes).

**Discussion:**

This is the first randomized clinical trial protocol to assess the effect of an exercise protocol that was designed specifically for the foot-and-ankle complex on running-related injuries to the lower limbs of long-distance runners. We intend to show that the proposed protocol is an innovative and effective approach to decreasing the incidence of injuries. We also expect a lengthening in the time of occurrence of the first injury, an improvement in foot function, an increase in foot muscle mass and strength and beneficial biomechanical changes while running and walking after a year of exercising.

**Trial registration:**

Clinicaltrials.gov Identifier NCT02306148 (November 28, 2014) under the name “*Effects of Foot Strengthening on the Prevalence of Injuries in Long Distance Runners*”. Committee of Ethics in Research of the School of Medicine of the University of Sao Paulo (18/03/2015, Protocol # 031/15).

**Electronic supplementary material:**

The online version of this article (doi:10.1186/s12891-016-1016-9) contains supplementary material, which is available to authorized users.

## Background

Human performance, particularly in one of the most popular sports activities such as running, is typically influenced by the state of the musculoskeletal system, either by the level of training and conditioning of the biological structures, or by the aging process. Although popular worldwide due to its low cost, versatility, convenience [1], and health benefits to people of all ages [2], running is associated with a high prevalence of lower extremity injuries (between 19.4 and 79.3 %) [3]. The occurrence of injuries limits the intended benefits by inducing changes in practice habits [4] or temporary or even permanent cessation of running. In addition, injuries lead to increased costs due to medical treatment and/or work absence [5].

The understanding of risk factors associated with these injuries, particularly the intrinsic factors, can provide important benefits for runners. Among these intrinsic factors, those that are noteworthy include biomechanical factors and muscle functionality of the lower extremities, particularly the feet. A systematic review by van der Worp et al. [5] included 11 high-quality longitudinal studies and concluded that alterations in the biomechanical force distribution patterns, amount of training, history of previous injuries, increased index of the navicular drop, and the misalignment of the ankle, knee, and hip are among the main intrinsic risk factors for running-related injuries. In addition, extrinsic factors such as the training surface and the type of footwear are also relevant risk factors [5]. It is noteworthy that out of these seven diverse risk factors, two are related to the foot-ankle complex, demonstrating the importance of maintaining the health and functionality of its musculoskeletal structures to prevent injuries. It is also believed that any biomechanical alteration in the musculoskeletal system, in particular the foot-ankle complex, broadly influences a runner’s functionality, predisposing him/her to a lesser or greater extent to injuries, in addition to the possibility of compromising his/her quality of life [2, 6].

The foot has a complex structure that can perform a broad variety of functions in different postural and dynamic tasks [7, 8]. This versatility can only be achieved through its unique arch-shaped architecture and its powerful intrinsic and extrinsic muscular activity, which is responsible for the maintenance and control of foot arches, postural corrections during disturbances, and torque generation during body displacement [9, 10]. Even with this unique and specialized structure, a high prevalence of injuries associated with running practices occurs in this complex. Among the most common hypotheses used to explain this high prevalence are factors such as the excessive ankle/foot pronation in the stance phase of running [11], the lowering of the medial longitudinal arch due to navicular drop [12, 13], the alteration of rigidity of the plantar arches [14], and the increase in impact and acceleration of the tibia during running [15].

Evidence suggests the importance of the intrinsic foot musculature, showing that fatigue can cause a significant increase in pronation, which is evaluated by the navicular drop [12]. In addition, weakness may be a risk factor for falls in the elderly population [16]. Therefore, it is understandable that the specific training of foot [13, 17] and ankle muscles [18–20] is an important tool that improves functions and functionalities of the lower extremities, as has been shown in recent studies [13, 19–21].

In one of those studies, the unsupervised practice of a single exercise for the feet (short-foot exercise) four times a week promoted a decrease in the navicular drop, an increase in the medial longitudinal arch index, and an increase in the functionality quality of the intrinsic foot muscles in asymptomatic individuals [13]. These results were maintained 1 month after the training had been completed. Although the results of Mulligan and Cook [13] are promising, they only measured the foot function in static conditions and the unsupervised practice of an isolated exercise for 4 weeks may not have been sufficient to cause a transfer of the static gains for a more dynamic task where the foot would be more robustly utilized, according to the star excursion balance test. In contrast, one study compared two groups: one group performed a 4-week period of short-foot exercises, including 100 repetitions for five seconds each, and the second group performed a 4-week period of towel-curl exercises with the same amount of exercise [20]. This controlled study showed that both groups exhibited decreased displacement of the centre of pressure during the modified star excursion test. Therefore, a load increase in the same exercises used by Mulligan and Cook [13] resulted in positive effects for postural control.

The same short-foot exercise was practiced by individuals with flat feet in a randomized controlled trial to investigate its effect on the use of foot orthoses [17]. The protocol consisted of three to five sets of exercises with five repetitions each, twice a day, for 8 weeks. In both study groups, the isometric force and the transversal section area of the abductor hallucis muscle were increased after the interventions, with a significant increase in the group that used orthoses during exercises. These results demonstrated that even in structurally unfavourable conditions, exercise for the foot muscles leads to important strength gains. It is noteworthy that even with a well-planned intervention, the lack of a control group and the evaluation of the muscle strength alone limit the study conclusions. In addition, the study did not take into account the potential clinical and functional changes of the plantar arches, as performed by Goldmann et al. [19]. This group of researchers investigated the effects of the hallucis flexors strengthening in the kinetic and kinematic of foot and ankle during walking, running, and vertical jumping among university athletes. Training of the experimental group consisted of isometric contractions of the hallucis flexors at 90 % of the maximum voluntary contraction using a dynamometer four times a week for 7 weeks. The authors observed a significant increase in the performance of vertical jumping and extensor and flexor momentum of the metatarsal-phalangeal joint and a gain of 60 to 70 % in the strength of the hallucis flexors. This study shows that the flexor muscles of the foot respond in a quick and intense manner to training; even for simple training, the strengthening of the muscles in question results in global kinematic and kinetic alterations. It would still be interesting to determine how long these gains would last after the completion of the intervention and whether more elaborate training, involving more muscles and different postures and loads, would alter the study outcome, especially with regard to foot biomechanics during locomotor tasks.

The understanding of the effects of a therapeutic approach focused on the foot biomechanics of walking and running, on the strength and functionality of lower extremity muscles will contribute to the adoption of more effective therapeutic and preventive strategies for runners. However, no evidence exists that supports the efficacy of the therapeutic exercises already used and recommended for the health of the feet [7, 17, 19, 20, 22] with regard to preventing recurrent injuries in long-distance runners. However, one research protocol aims to assess the effects of ankle and hip muscle strengthening and functional balance training on running mechanics, postural control, and injury incidence in novice runners with less than 1 year of running experience but without focusing on the intervention of intrinsic and extrinsic muscles of the feet [23].

Therefore, a controlled and randomized clinical trial would determine whether these interventions are efficacious by using the incidence of running-related injuries as the primary outcome and following both intervention and control subjects during a period of time equal to or greater than 1 year (the period during which the incidence and prevalence of these injuries are reported) [4, 16, 24–27].

It is important to highlight that rehabilitation programs rarely include the intrinsic muscles of the feet in their therapeutic protocols. The present proposal uses a new paradigm in which the focus of training and preventive interventions in runners is a “ground-up” approach rather than the traditional "top-down" approach, which focuses on the hip strengthening. This new approach, advocated by Baltich et al. [23], will seek to improve the function of the ankle-foot complex, which is directly associated with the absortion and transmission of body forces to the ground and vice-versa during running.

### Hypotheses

Our hypotheses are that the therapeutic exercise protocol for the foot-ankle as practiced by long-distance recreational runners for 1 year will:

- H 1. Reduce the incidence of running-related injury in the lower limbs,
- H 2. Lengthening the time for the occurrence of the first running-related injury in the lower limbs,
- H 3. Increase intrinsic foot muscle strength,
- H 4. Increase foot muscle cross-sectional area and volume,
- H 5. Improve foot health and functionality status,
- H 6. Reduce dynamic strain on the foot’s longitudinal arch during running and walking, and
- H 7. Produce beneficial biomechanical changes during running that denote an improvement in the mechanical efficiency of absorbing loads and propelling the body while walking and running. Such changes would include an increase in the ankle range of motion in the sagittal plane and increases in 1) ankle extensor moment and power and 2) knee extensor moment and power during the second half of the stance phase.

Our aim is therefore to investigate the effects of a "ground-up" therapeutic approach focused on the foot-ankle for 1 year as they relate to 1) the incidence of running-related injuries in the lower limbs of long-distance runners, 2) time of occurrence of the first injury, 3) foot health and functionality, 4) strength of the intrinsic foot muscles; 5) foot muscle trophism, 6) dynamic foot arch strain and 7) lower-limb biomechanics during walking and running.

## Methods/Design

### Overview of the research design

A randomized, prospective controlled and parallel trial with blind assessment is designed to study the effects of a "ground-up" therapeutic approach focused on the foot-ankle concerning the incidence of running-related injuries to the lower limbs of long-distance runners. This trial has an allocation ratio of 1:1. Its framework is exploratory to gather preliminary information on the intervention of conducting a full scale trial. The trial follows all recommendations established by SPIRIT [28].

Long-distance recreational runners are recruited from the vicinity of the city of São Paulo and referred to a physical therapist, who performs the group allocation. The participants are then referred to another physical therapist, who performs the initial blind assessment. All runners allocated to the intervention group (IG) participate in a protocol of therapeutic exercises for the foot-ankle complex for 8 weeks, with one session per week supervised by a physical therapist and three sessions per week remotely supervised by web-enabled software [29]. They receive access to the web software on the first day and use it for 8 weeks. After the 8-week period, the IG runners will continue exercising for 10 more months, supervised only by the web software three times a week. The runners allocated to the control group (CG) do not receive any intervention training, but receive a placebo stretching exercise program.

All runners will be assessed at baseline and 2 months (end of intervention). They are then assessed twice more for follow-up purposes, at 4 and 12 months after the baseline. Assessments will concern the incidence of running-related injuries (primary outcome), and all other secondary outcomes.

**Fig. 1:**
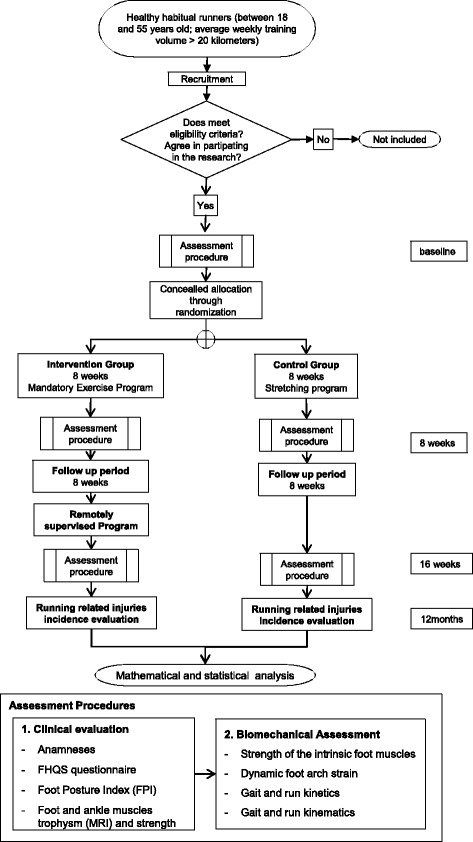
Flow chart of the study’s design

The design and flowchart of the protocol are presented in Fig. 1. The assessments are performed at the Laboratory of Biomechanics of Human Movement and Posture (LaBiMPH) at the Physical Therapy, Speech and Occupational Therapy department of the School of Medicine of the University of São Paulo, São Paulo, Brazil.

### Participants and recruitment

This study is currently recruiting patients (study start date: April 2015)

The eligibility criteria for the volunteer runners are:

- aged between 18 and 55 years old
- at least 1 year of running experience
- a weekly training distance greater than 20 km an less than 100 km as their main physical activity
- within 2 months prior to baseline assessment, lack of any lower limb musculoskeletal injury or pain that might lead to stopping running practice
- no prior experience within the last year of isolated foot and ankle strength training
- not receiving any physical therapy intervention
- no history of using minimalist shoes for running practice
- no prior experience of barefoot running

Runners are not selected if they have other neurological or orthopedic impairments (such as congenital foot malformations, stroke, cerebral palsy, poliomyelitis, rheumatoid arthritis, prosthesis or moderate or severe osteoarthritis), major vascular complications (venous or arterial ulcers), diabetes mellitus, sequelae from poorly healed fractures or prior lower-limb surgeries.

These runners may use the running technique of fore-, mid- or rear-foot ground contact, which will be classified by the strike index, according to Cavanagh and Lafortune [30].

One hundred and eleven (111) runners will be recruited by radio advertisements, print media and running association groups at their site of practice around the city of São Paulo. The potential subjects will be interviewed by telephone and, when selected, assessed in the laboratory to confirm all the eligibility criteria. This first laboratory assessment represents the baseline condition (blind assessment).

The runners allocated to the IG will be treated during their locally supervised session at the Physical Therapy Department in an ambulatory setting that assists all the physical therapy treatments of the Department, providing a reliable therapeutic environment for the intervention.

### Randomization, allocation and blinding

The randomization schedule was prepared using Clinstat software [31] by an independent researcher (Researcher 1) who was not aware of the numeric code for the CG and IG groups. A numeric block randomization sequence will be kept in opaque envelopes.

After the runners’ agreement to participate and assignment in the research, the allocation into the groups will be made by another independent researcher (Researcher 2), who also will be unaware of the codes. Only the physiotherapist (Researcher 3) responsible for the locally supervised training knows who is receiving the intervention. Researcher 3 will also be responsible for the remote monitoring of the training by web software [29] and telephone. One physiotherapist (Researcher 4), who will also be blind to the treatment allocation, will be responsible for all clinical, functional and biomechanical assessments. Both physiotherapists (researchers 3 and 4) will be blind to the block size used in the randomization procedure.

To guarantee the blindness of researcher 4, before each evaluation, runners will be instructed not to reveal whether they are in the CG or IG; their questions should be asked only to the physiotherapist in charge of web software [29] and local training (Researcher 3).

The trial statistician will also be blind to treatment allocation until the main treatment analysis has been completed.

### Treatment arms

The CG runners will receive a 5-min placebo routine of warm-up and muscle-stretching exercises to be performed immediately before every running practice during their 8-week study (Additional file 1: Table S3).

The IG runners will receive a therapeutic foot-ankle exercise protocol for strengthening and improving functionality under the supervision of a physiotherapist (Researcher 4) once a week for 8 weeks, and a series of foot-ankle exercises to be performed under remote supervision through web software [29] three times a week for the full 1-year length of the study (1 year). Both locally (Additional file 1: Table S1) and remotely supervised therapeutic routines (Additional file 1: Table S2) will take from 20 to 30 min. In particular, the remotely supervised practice will be preferentially performed at home; the web software includes written descriptions, photos and videos of each exercise.

Each week, IG runners will be requested to evaluate the subjective effort of each exercise’s performance using a score of 0 to 10 either with the web software [29] or to the physiotherapist during locally supervised practice. If the effort score ranges from 0 to 5 and the runner’s performance of each exercise is found adequate during the supervised session by the physiotherapist, the exercises will increase in difficulty according to the progression chart in Additional file 1: Table S1 and Table S2. If the effort score ranges from 6 to 7, the exercise will not increase in difficulty and no progression would be done on that exercise. Thus, the runner remains in the same exercise progression until he/she scores 0 to 5 in each particular exercise. Finally, if an IG runner reports a score from 8 to 10, the exercise will decrease in difficulty, if possible, until the subject is able to perform it without pain or discomfort.

### Assessments

A physiotherapist (Researcher 3) who is blind to group allocation will perform all assessments. Each assessment will consist of taking a clinical history of personal details, anthropometry, running practice details (years of practice, weekly frequency and volume, usual shoe and training surface, number of races and whether the runner trains with a running coach), previous orthopedic surgery, other physical activity practiced regularly (previous to running practice or simultaneously with running) and an injury history concerning the most important risk factors previously published [3, 32, 33].

A foot-health status questionnaire [34] will be used to characterize foot health and functionality. We will use a Brazilian-Portuguese version (FHSQ-BR) translated and validated by Ferreira et al. [35]. This instrument is divided into three sections. Section I evaluates foot health in four domains: foot pain, foot function, footwear and general foot health. Section II evaluates general health in four domains: general health, physical activity, social capacity and vigour. Sections I and II are composed of questions with answer options presented in affirmative sentences and corresponding numbers. Section III collects general demographic data of the individuals [36]. We will not use the scores from Section III. Each domain scores from 0 to 100 points, where 100 is the best condition and 0 the worst.

We will access variations in foot posture of the runners using the Foot Posture Index (FPI) [36]. The FPI is a six component measures that allows multiple segment evaluation of foot posture on a static measurement and requires that subjects stand in their relaxed stance position looking straight ahead while the assessment is in process. The assessment consists on the (1) palpation of the talar head, (2) observation of supra and infra malleolar curvature, (3) observation of the calcaneal frontal plane position, (4) observation of the bulging in the region of the talo-navicular joint, (5) observation of the height and congruence of the medial longitudinal arch and (6) presence of abduction or adduction of the forefoot. Scores reaching from -12 to +12 and normative values are presented on the literature.

Subjects will then be assessed for intrinsic foot muscles strength, lower-limb running kinematics and kinetics, and dynamic foot-arch strain. The feet of 30 % of the participants in each group (41 participants) will be imaged by magnetic resonance imaging (MRI) to assess trophism and strength of the foot intrinsic muscles; this will be scheduled for the same week of each subject’s baseline measurements.

After baseline assessment, all subjects will be scheduled for two follow-ups assessments, one at 8 weeks and the other at 16 weeks. They will maintain contact with the Researcher 3 through the follow-up period by the web software [29], e-mail and telephone.

#### Running-related injuries

Running-related injuries will be assessed initially at the baseline and will be assessed continually throughout the study by the web software [29]. The definition of running-related injury was set according to the study of Macera et al. [4]. They stated that any musculoskeletal pain or injury that was caused by running practice and that induces changes in the form, duration intensity or frequency of training for at least 1 week will be considered a running-related injury. Only lower-limb injuries will be accounted during the 12-month period after the baseline assessment; both the incidence and time of occurrence of the first injury will be analyzed.

If any subject presents a new injury during his or her participation in the study, the injury will be accounted for and the intervention or placebo intervention will be discontinued, even though all subjects will still keep being followed for the completion of the study.

#### Isometric intrinsic foot muscles strength

Strength of the foot’s intrinsic muscles will be assessed in trials using a pressure platform (EMED: Novel, Germany) on which the subjects will place their dominant foot while standing with knees extended. They will push down as hard as possible using only their hallux and toes, particularly the metatarsophalangeal joints and not the hallux interphalangeal joint. A physiotherapist will determine whether the subject lifted the heel, and inspect fluctuations in the line of gravity and trunk posture during each trial. If any changes are observed in the line of gravity or positioning of the heel or trunk, the trial will be excluded. Three trials will be completed on each foot (left and right) according to Mickle et al. (2006) [37]. Maximum force will be normalized by body weight and analyzed for hallux and toes areas separately.

#### Foot muscle trophism and strength

One indirect method of measuring foot strength is through MRI, which, combined with other techniques, offers good reliability and a way to follow changes in muscular volume [38]. In addition, MRI can facilitate understanding the etiology of running-related injuries and rehabilitation of the foot-ankle complex [39].

The MRI of the foot will be performed with a 1.5 T system. Foot images will be acquired by the same technician using a coil of four channels positioned in the magnetic centre. Participants will be placed in supine position with the ankle at 45° of plantar flexion inside the coil. Images will be acquired in the frontal, sagittal and transverse planes to confirm the position of the feet, and the subject will be repositioned if necessary. T1-weighted images of the entire foot length will be acquired perpendicular to the plantar aspect of the foot using a spin-echo sequence (repetition time = 500 ms, echo time = 16 ms, averages = 3, slice thickness = 4 mm, gap between slices = 0 mm, field of view = 120 × 120 mm, flip angle = 90°, matrix = 512 × 512) [39]. The set of images will cover the distance between the most proximal and most distal images in which every intrinsic foot muscle is visible.

To assess changes in the cross-sectional area (CSA) and volume of the intrinsic foot muscles, 30 % of the subjects from each group will have MRI of the foot at three times: baseline, 8 weeks and 16 weeks.

The CSA will be measured by ImageJ planimeter software [40]. Following, Miller et al. [14] for each muscle at each slice and muscle volume will be calculated by multiplying the CSA of all slices for a muscle by their linear distance (4 mm) and adding these volumes.

#### Walking and running biomechanics

To ensure maximum reliability, all biomechanical testing sessions will be completed by the same researcher.

Gait and running kinematics will be acquired using three-dimensional displacements of passive reflective markers (10 mm in diameter) tracked by nine infrared cameras at 100 Hz (OptiTrack FLEX: V100, Natural Point, Corvallis, OR, USA) [41, 42]. Some 14 markers will be placed on the right subject’s foot according to Leardini’s protocol [43]. Extra markers will be placed at the medial knee joint line, lateral knee joint line and bilaterally at the iliac spine antero-superior, superior aspect of the greater trochanter, and sacrum. These markers will be used to determine relative joint centres of rotation for the longitudinal axis of the foot, ankle and knee. The extra markers from the medial aspect of the knee joint line will be removed during the dynamic trial. In addition, three non-collinear reflective markers will be fixed at two technique clusters. One of the clusters will be placed in the lateral thigh and the other over the shank.

The laboratory coordinate system will be established at one corner of the force plate and all initial calculations will be based on this coordinate system. Each lower-limb segment (shank and thigh), will be modelled based on surface markers as a rigid body with a local coordinate system that coincides with the anatomical axes. Translations and rotations of each segment will be reported relative to the neutral positions defined during the initial static standing trial. All joints will be considered to be spherical (i.e., with three rotational degrees of freedom). The foot will be modeled according to Leardini et al. [43]. That is, the calcaneus, mid-foot and metatarsus are considered rigid bodies and the longitudinal axis of the first, second and fifth metatarsal bones and proximal phalanx of the hallux will be tracked independently.

Ground reaction forces will be acquired by a force plate (AMTI OR-6-1000, Watertown, MA, USA) with a sampling frequency of 1 kHz embedded in the centre of the walkway. Force and kinematic data acquisition will be synchronized and sampled by an A/D card (AMTI, DT 3002, 12 bits).

The subjects will go through a habituation period before the data acquisition to establish confidence and comfort in the laboratory environment, and to ensure appropriate movement velocity. To assess lower-extremity running mechanics, subjects will perform 10 valid over-ground walking trials and 10 valid over-ground running trials at a constant velocity (9.5 km/h to 10.5 km/s); these will be monitored by two photoelectrical sensors (Speed Test Fit Model, Nova Odessa, Brazil).

The automatic digitizing process, 3D reconstruction of the markers’ positions and filtering of kinematic data will be performed using AMASS software (C-motion, Kingston, ON, Canada). Kinematic data will be processed using a zero-lag second-order low-pass filter with cutoff frequencies of 6Hz for walking and 12 Hz for running**.** Ground reaction force data will be processed using a zero-lag low-pass Butterworth fourth-order filter with cutoff frequencies of 50Hz for walking and 200 Hz for running.

A bottom-up inverse dynamics method will be used to calculate the net moments in the sagittal and frontal planes of the ankle and knee joints using Visual3D software (C-motion, Kingston, ON, Canada). The human body will be modeled by three linked segments (foot, shank and thigh) and the inertial properties will be based on Dempster’s standard regression equations. The moment of inertia and location of center of mass will be computed assuming the thigh and shank segments as cylinders.

Calculation of all variables will be performed using a custom-written MATLAB function (MathWorks, Natick, MA, USA). Data of only one lower limb (randomly chosen) per subject will be analyzed and compared.

The following ankle kinematic variables will be analysed: maximum dorsiflexion at foot contact, maximum plantarflexion, maximum dorsiflexion at the toe-off and dorsiflexion range of motion (ROM) in the sagittal plane during the stance phase. The knee kinematic variables are: maximum flexion at foot contact, maximum extension, maximum flexion in the stance phase, ROM on sagittal plane, maximum abduction and adduction in the stance phase. The foot kinematic variables are: elevation/drop of the longitudinal arch angle and of the first, second and fifth metatarsal bones; rearfoot to forefoot rotation; transverse plane angle between first and second metatarsal bones and between second and fifth metatarsal bones; and maximum inversion and eversion of the calcaneus (frontal plane).

The ankle and knee kinetic variables to be analysed are net ankle and knee moments normalized by body weight times height and power normalized by body weight in the sagittal plane. The ground reaction force variables will be normalized by body weight and are as followings: first peak force (body weight – BW), second peak (BW), loading rate 80 [N/ms], defined as the force rate between 20 and 80 % of the contact of the foot with the ground during the first peak; loading rate 100 [N/ms], as determined by the force rate between 0 and 100 % of the first peak and push-off rate [N/ms], as defined as the rate of the second peak force, between the minimal values until the second peak.

#### Dynamic longitudinal foot arch strain

The dynamic longitudinal foot arch strain will be measured according to Liebermann et al. [44]. The measurement involves navicular height (NH), which is the minimum distance from the navicular tuberosity relative to the line formed by the first metatarsal head and the medial process of the calcaneus. These three landmarks form a plane and NH is independent of rear-foot inversion or eversion. Arch strain can also be quantified by fitting a parabola to markers (with the navicular head as the vertex) and then measuring the average curvature at 100 points evenly spaced along the curve.

### Outcome measurements

The primary outcome measurement will be the incidence of running-related injuries in the lower limbs accounted at the end of 12 months of study.

The secondary outcomes will be: 1) the time of the occurrence of the first injury along the study period (time to event); 2) foot health and functionality (change from baseline); 3) foot, ankle and knee kinematics, ankle and knee joint moments, and knee and ankle power during walking and running (change from baseline); 4) strength of the intrinsic foot muscles (change from baseline); 5) foot muscle trophism (change from baseline); and 6) dynamic foot arch strain (change from baseline).

### Interventions

Runners allocated to the IG will receive a foot-ankle therapeutic exercise protocol for strengthening and improving functionality. Part of the exercise protocol (12 exercises) is to be performed once a week under the supervision of a physiotherapist for 8 weeks (Additional file 1: Table S1). And a series of eight foot-ankle exercises is also to be performed three times a week remotely supervised by web software [29] (Additional file 1: Table S2) for the full 1-year completion time of the study. Each session, whether supervised locally or remotely, lasts 20 to 30 min. The therapeutic exercise protocol is described in details in Additional file: 1 Table S1 and S2.

Gradual and progressive difficulty will be offered to the runner, respecting any limitation due to pain, fatigue and/or decrease in performance during execution. The runners in the IG will access the web software [29] daily, entering their data regarding performance of the foot exercise training and ranking their level of difficulty in each exercise from 0 to 10.

During the locally supervised sessions, the physiotherapist will focus on proper alignment of the foot-ankle segments, especially if the runner has difficulty in maintaining it, in a way that allows no movement compensations.

Runners allocated to the CG will receive a 5-min placebo warm-up and muscle stretching exercise routine (Additional file 1: Table S3) that they are to perform for 8 weeks immediately before each running practice. This placebo training can also be assessed and followed through the web software [29]. The stretching exercises are described in detail in Additional file: 1 Table S3. We hypothesized that a warm-up combined with muscle stretching exercises would not have any effect on foot muscular strength and functionality, lower extremity biomechanics or injury prevention.

Both groups will access the web software [29] daily, entering their running practice data (daily training duration and volume) and information concerning the occurrence of any injury event.

The discontinuation criteria for the exercises during any session includes cramps, moderate to intense pain, fatigue or any other condition that exposes the runner to any discomfort.

The discontinuation criteria for the training includes an occurrence of a running-related injury in the lower limbs.

If any subject fails to access the web software [29] for three consecutive weeks without explanation, or fails to attend the locally supervised training three consecutive times, that subject will be terminated from the study.

To improve adherence, several actions will be performed by the researchers in the web software [29]. Data regarding the subjects running practice, such as training volume, time of practice and occurrence of injuries, will be reported to the web software, which will summarize it and make it viewable in the users’ area. In addition, for the duration of the study, runners' responses in the web software concerning their foot-ankle exercise practice and running training will be stored and be accessible to the researchers and subjects at any time. If any subject fails to log in to the web software for more than five consecutive days, an e-mail will be automatically be sent, asking the subject to log in to his or her account and report data on the training (or lack of it) for the past week. The physiotherapist responsible for the therapeutic protocol will make phone contact with subjects who fail to attend to any of the weekly locally supervised sessions. They will also make phone contact with subjects who do not respond to e-mail reminders from the web software. Subjects will also be contacted by personal phone calls if data they reported on the web software is found to be inconsistent [45].

After the period of intervention and after 12 weeks of follow up all runners will be questioned about their satisfaction to the training protocol with one question (Did you enjoy doing the exercises?) with three answer possibilities (No; A Little; A lot). To avoid evaluation bias, runners will answer this question secretly through an online-unidentified form sent to their e-mail. Runners will be informed about the anonymity and this form will only be accessed after completion of the study.

For the duration of the trial, subjects will be advised not to engage in any new physical activity or preventive training protocols for the foot and ankle. If any subject cannot avoid such behavior, he or she must report this situation during web software [29] access. Concomitant care, such as physical therapy, acupuncture or other conventional medical care, will not be permitted except for runners who are injured during the study. At the end of 12 months, CG participants that are interested will receive access to the software for the foot exercise protocol.

### Sample size and statistical analysis

The sample size calculation was made using an effect size of 0.28 (proportion), considering the categorical primary outcome variable, which is the incidence of running-related lower-limb injuries [33]. A sample size of 101 runners is needed to provide 80 % power to detect a moderate effect difference between the highest and lowest group injury incidence medians, assuming an alpha of 0.05 and a *χ*^2^ (chi-squared test) statistical design – contingency tables (df = 1) [46]. Assuming a 10 % dropout rate during the study, a sample size of 111 runners is needed.

The statistical analysis will be based on intention-to-treat analysis, and mixed general linear models of analysis of variance for repeated measure will be used to detect treatment-time interactions (α = 5 %). The outcome measures will be compared at baseline, 2, 4 and 12 months. Effect sizes (Cohen´s d coefficient) will also be provided between baseline and 2 months and between 2 months and follow-up (4 and 12 months), if the intervention shows any treatment effect. The missing data will be treated by imputation methods depending on the type of the missing data we will face: missing completely at random, missing at random, or missing not at random [47].

## Discussion

This clinical trial will provide important data on foot-training effectiveness, its influence on the incidence of injuries and its efficacy on strengthening the muscles of the foot-ankle complex. It will also facilitate the identification of risk factors and biomechanical mechanisms involved in injury processes and prevention. We also intend to contribute new evidence that could be used as a guide for further studies on biomechanical changes in dynamic tasks resulting from the strengthening of the foot-ankle complex.

The few existing clinical trials that have proposed exercise protocols to reduce the incidence of runners’ injuries have not included the incidence of injury as a primary outcome. They also have had short follow-up periods and usually failed to follow the subjects’ adherence to the program and the correctness of exercise performance throughout the study [13, 17, 19, 20]. In contrast, this trial has the incidence of running-related injuries as a primary outcome, will have a long period of follow-up (12 months), proposes an intervention training protocol with several exercises that are easy to perform with short durations for each session (20–30 min) and does not require subjects to be continuously supervised by a health professional. In addition, it utilizes open-access web software [29] that will support adherence control.

We understand that the number of MRIs that we are performing (on 30 % of the subjects) is limited and might prevent a broad conclusion about changes in intrinsic foot muscle cross-sectional area (CSA) and volume.

Running-related injuries in this population cause interruptions and abandonment of physical activity. They also could lead to the development of chronic injury that would prevent the practice of other sports and hence frustrate the individual’s pursuit of a healthy lifestyle. Runners are constantly looking for ways to remain free from injury and the information they receive from coaches or media is often conflicting and varied [48]. Our protocol has the potential to change the course of this vicious cycle experienced by long-distance runners.

If our hypothesis that such an exercise protocol reduces the incidence of running-related injuries to long-distance runners is confirmed, it could be easily incorporated into their warm-up routine prior to running practice.

## Ethics approval and consent to participate

This trial was approved by the Ethics Committee of the School of Medicine of the University of São Paulo (Protocol number n°031/15). Additionally, this trial is registered in ClinicalTrials.gov (a service of U.S. National Institutes of Health) Identifier NCT02306148 (November 28, 2014) under the name “*Effects of Foot Strengthening on the Prevalence of Injuries in Long Distance Runners”.* All runners will be asked for written informed consent according to the standard forms and the researcher 4 will obtain them.

## Consent to publish

Written informed consent for publication of all images was obtained from the models.

## Availability of data and materials

All personal data from potential or enrolled runners will be maintained confidential before, during and after the trial by encoding participant’s name. All data access and storage are in keeping with National Health and Medical Research Council guidelines, as approved. All files will be available from the database published at figshare.com. All important protocol amendments will be reported to investigators, review boards and trial registration by the Researcher 3.

## Additional file

Additional file 1:
**Table S1.** Exercises included in the supervised sessions by a physiotherapist. **Table S2.** Exercises included in the remotely supervised sessions in the web software. **Table S3.** Warm up and stretching exercises - Control group. (DOCX 2425 kb)

## Abbreviations

CG: Control group
CSA: Cross-sectional area
FHSQ-BR: Foot-health status questionnaire - Brazilian-Portuguese version
FPI: Foot Posture Index
IG: Intervention group
MRI: Magnetic resonance imaging
NH: Navicular height
