# Supplementary material for: Protocol for evaluating the effects of a therapeutic foot exercise program on injury incidence, foot functionality and biomechanics in long-distance runners: a randomized controlled trial
Source: BMC Musculoskelet Disord. 2016 Apr 14;17:160. doi: 10.1186/s12891-016-1016-9 (PMC4831173; doi:10.1186/s12891-016-1016-9)
Supplement: Additional file 1: — Table S1. Exercises included in the supervised sessions by a physiotherapist. Table S2. Exercises included in the remotely supervised sessions in the web software. Table S3. Warm up and stretching exercises - Control group. (DOCX 2425 kb) [file 12891_2016_1016_MOESM1_ESM.docx]

**Additional file 1**

**Table S1 -** Exercises included in the supervised sessions by a physiotherapist.

| **Name** | **Execution** | | | **Training Volume** | | **Progression** | | **Progression Parameter** | | **Approximate Duration** |  |
| --- | --- | --- | --- | --- | --- | --- | --- | --- | --- | --- | --- |
| Massage  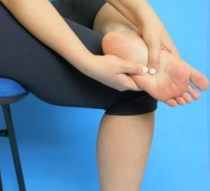 | Sitting, with leg crossed over the other, massage the sole of your feet with both hands, for 20 seconds. Rub your foot in a circular motion using your thumb. Do the same on the other foot. | | | 1 set of 20 seconds each foot | | - | | - | | 40 Seconds |  |
| Toes manipulation  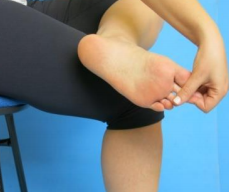 | Sitting, with leg crossed over the other, hold each toe and slowly spin side to side, like a screw. Do with all toes. | | | 1 set of 10 times each finger | | - | | - | | 1 minute |  |
| Rubber ball slide  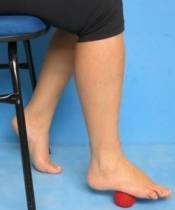 | Slowly slide your foot on the ball throughout the foot sole from the heel to the fingertips. | | | 1 set of 30 seconds each foot | | - | | - | | 1 minute |  |
| Feet tapping  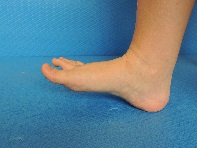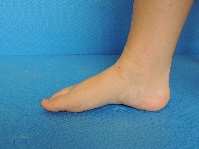 | With the heel fixed, tap your foot as fast as possible. Starts seated on a chair, and do with both feet at the same time. After you learn, do the same tapping standing. | | | 1 set of 30 repetitions | | 1: 1x30 repetitions;  2: 2x30 repetitions;  3: 2x40 repetitions | | Being able to perform the set without pain or muscle cramp after the completion of the set. | | 1-2 minutes |  |
| Forefoot ascend  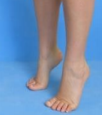 | Standing, ascend and descend on forefoot. Start standing, using both feet. Use a chair or table to keep balance. | | | 1 set of 30 repetitions | | 1: 1x30 repetitions;  2: 2x30 repetitions;  3: 2x40 repetitions | | Being able to perform the set without pain or muscle cramp after the completion of the set. | | 1-2 minutes |  |
| Invert/Evert asymmetric  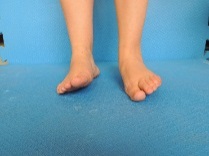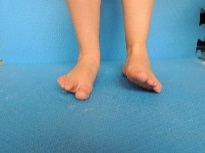 | Sitting, with 90 degrees of knee and ankle flexion, perform asymmetrical foot inversion (lifting medial side) and eversion (lifting lateral side). | | | 1 set of 10 repetitions maintaining each position for 1 second. | | 1: Sitting: 1x10 repetitions;  2: Standing: 1x10 repetitions ;  3: Standing 1x20 repetitions maintaining each position for 2 seconds. | | Being able to perform the set without pain or muscle cramp after the completion of the set, and without loss of balance. | | 1-2 minutes |  |
| Foot abduction  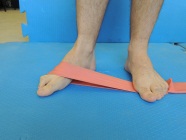  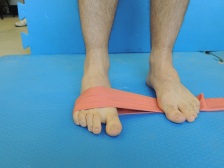 | Standing, using a resistance band around the forefoot, perform foot abduction and return to the original position | | 2 sets of 10 repetitions each foot | | 1: 2x10 repetitions;  2: 4x10 repetitions;  3: 6x10 repetitions. | | Being able to perform the set without pain or muscle cramp after the completion of the set. | | 1-6 minutes | |  |
| **Toes and ankle flexion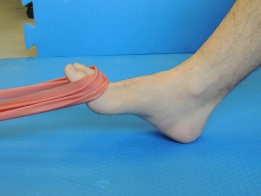**  **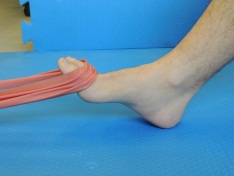** | Sitting posture, using a resistance band around the forefoot, perform ankle and toes flexion and return to the original position | | 1 sets of 10 repetitions each foot | | 1: 1x10 repetitions;  2: 2x10 repetitions;  3: 3x10 repetitions. | | Being able to perform the set without pain or muscle cramp after the completion of the set. | | 1-3 minutes | |  |
| **Grab and hold squeeze ball**  **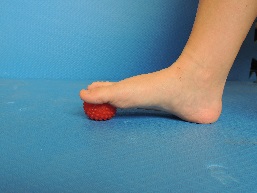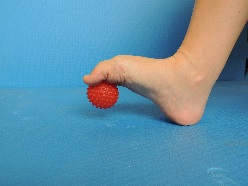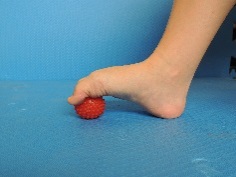** | Grab and hold a squeeze ball with all the toes, raise it from the floor and place it back to it’s original position. Always keep the heel fixed on the ground. | | 1 sets of 5 repetitions each foot holding the ball for 5 seconds | | 1: Sitting posture  1x5 repetitions;  2: Standing posture  2x5 repetitions;  3: Standing posture  3x5 repetitions. | | Being able to perform the set without pain or muscle cramp after the completion of the set. | | 2-6 minutes | |  |
| **Squeeze toes separators**  **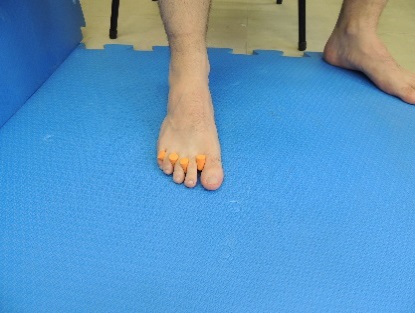**  **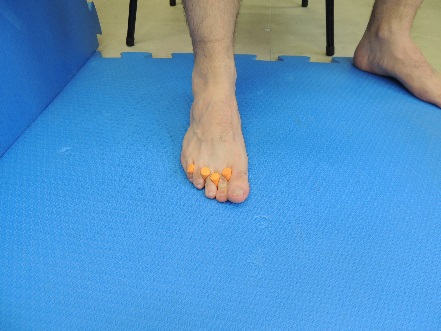** | | Sitting position, with 90 degrees of knee and ankle flexion, adduct and abduct, squeeze the toes separators for one second always keeping the heel fixed on the ground. | 1 sets of 10 repetitions each foot | | 1: 1x10 repetitions;  2: 2x10 repetitions;  3: 3x10 repetitions. | | Being able to perform the set without pain or muscle cramp after the completion of the set. | | 2-6 minutes | | |
| **Squeeze ball with little toes**  **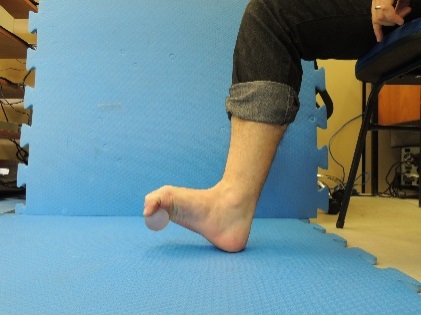**  **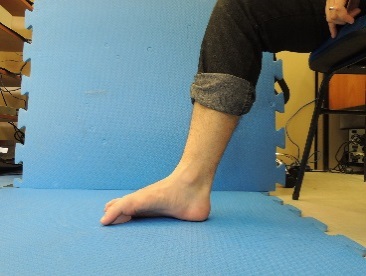** | | Grab and hold a squeeze ball with the metatarsophalangeal region and place it back to the starting position. | 1 sets of 5 repetitions each foot holding the ball for 5 seconds | | Progression requires raising squeeze balls hardness. | | Being able to perform the set without pain or muscle cramp after the completion of the set and being able to hold abduction for the stipulated time. | | 2 minutes | |  |
| **Toes Abduction/adduction**  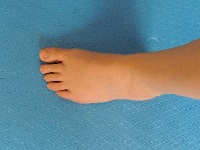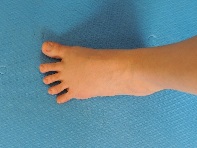 | | Sitting position, with 90 degrees of knee and ankle flexion, adduct and abduct toes holding each position for 2 seconds. | 1 sets of 10 repetitions each foot holding abduction for 2 seconds and adduction for 2 seconds. | | 1: Sitting posture  1x10 repetitions;  2: Standing posture  2x10 repetitions;  3: Standing posture  2x10 repetitions holding abduction/abduction for 5 seconds. | | Being able to perform the set without pain or muscle cramp after the completion of the set and being able to hold abduction for the stipulated time. | | 1-2 minutes | |  |
| **Short-foot exercise**  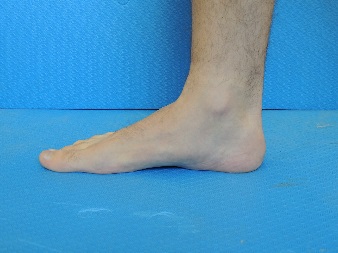  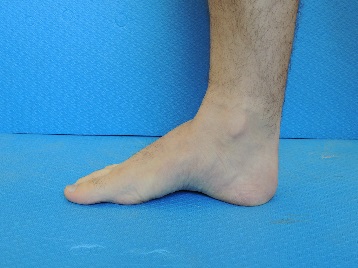 | Sitting, with 90 degrees of knee and ankle flexion, approximate the head of the first metatarsal toward the heel without toe flexion, “shortening” the feet. The forefoot and heel should not get off the ground. | | 1 set of 10 repetitions each foot, maintaining 5 seconds each contraction. | | 1: Sitting  1x10 repetitions;  2: Standing  1x10 repetitions;  3: Single leg stance  1x10 repetitions. | | Being able to perform the set without pain or muscle cramp after the completion of the set. | | 4-6 minutes | |  |
| **Plantar arch raise**  **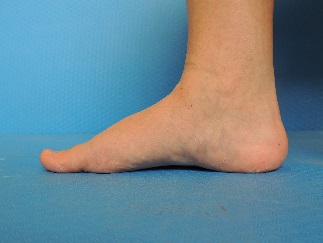**  **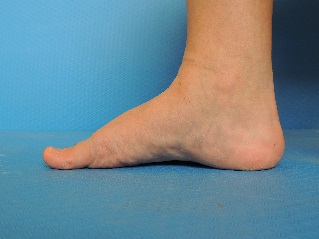** | Sitting, raise the plantar arch in an arch shape. The heel and fingertips should not get off the ground. | | 1 set of 10 repetitions each foot, maintaining 5 seconds each contraction. | | 1: Sitting  1x10 repetitions;  2: Standing  1x10 repetitions;  3: Single leg stance  1x10 repetitions. | | Being able to perform the set without pain or muscle cramp after the completion of the set. | | 4-6 minutes | |  |

**Table S2 –** Exercises included in the remotely supervised sessions in the web software.

| **Name** | **Execution** | **Training Volume** | **Progression** | **Progression Parameter** | **Approximate Duration** |
| --- | --- | --- | --- | --- | --- |
| **Massage**  **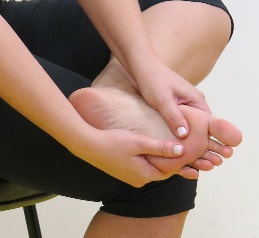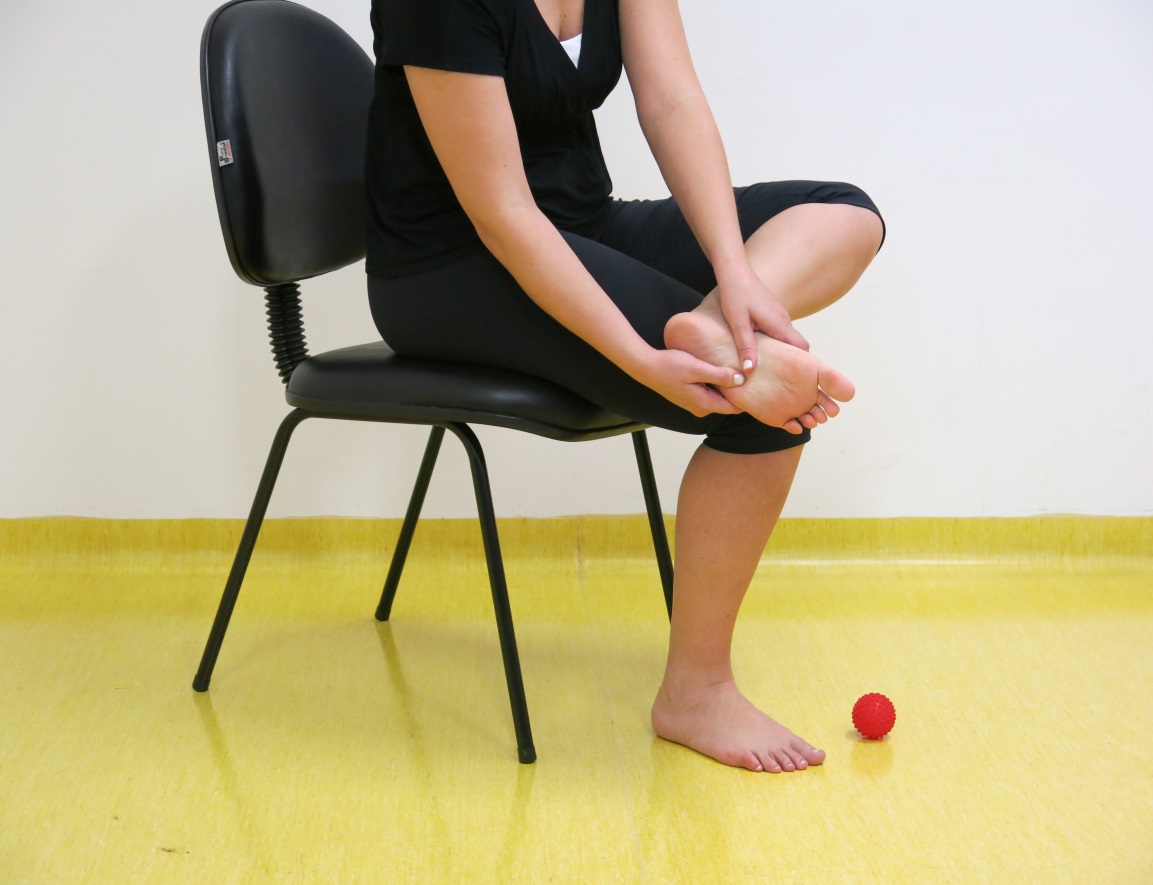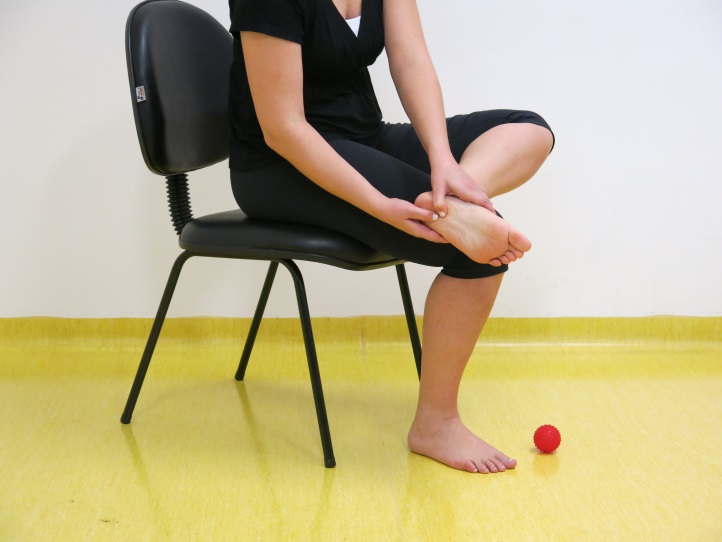** | Sitting, with leg crossed over the other, massage the sole of your feet with both hands, for 20 seconds. Rub your foot in a circular motion using your thumb. Do the same on the other foot. | 1 set of 20 seconds each foot | - | - | 40 seconds |
| **Toes manipulation**  **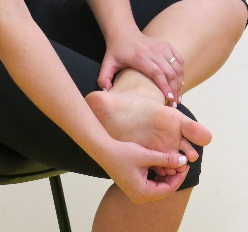** | Sitting, with leg crossed over the other, hold each toe and slowly spin side to side, like a screw. Do with all toes. | 1 set of 10 times each finger | - | - | 1 minute |
| **Feet tapping**  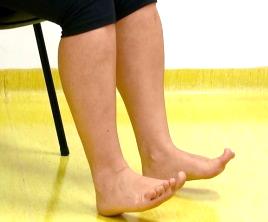 | With the heel fixed, tap your foot as fast as possible. Starts seated on a chair, and do with both feet at the same time. After you learn, do the same tapping standing. | 1 set of 30 repetitions | 1: 1x30 repetitions;  2: 2x30 repetitions;  3: 2x40 repetitions ; | Being able to perform the set without pain or muscle cramp after the completion of the set. | 1-2 minutes |
| **Forefoot accend**  **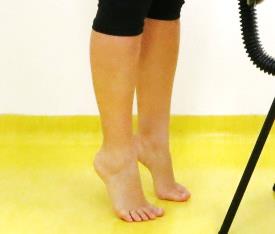** | Standing, ascend and descend on forefoot. Start standing, using both feet. Use a chair or table to keep balance. | 1 set of 30 repetitions | 1: 1x30 repetitions;  2: 2x30 repetitions;  3: 2x40 repetitions | Being able to perform the set without pain or muscle cramp after the fulfillment. | 1-2 minutes |
| **Invert/Evert symetric**  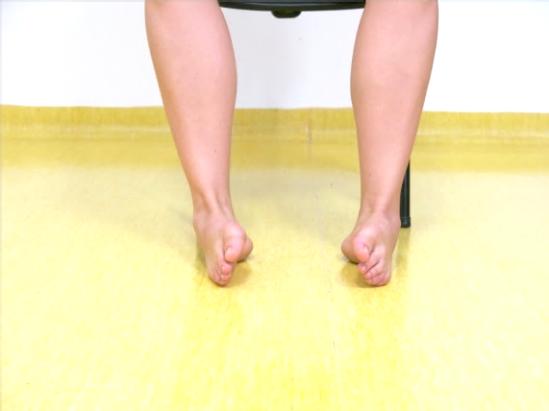  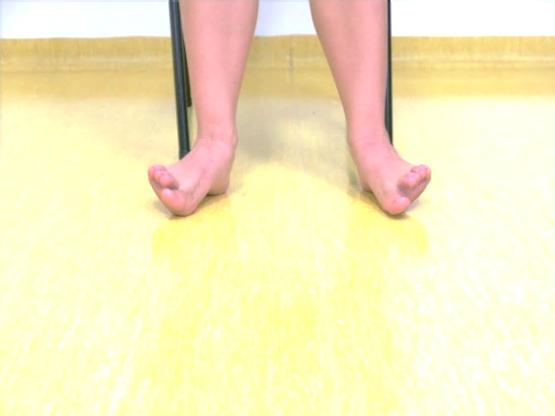 | Sitting, with 90 degrees of knee and ankle flexion, perform symmetrical foot inversion (lifting medial side) and eversion (lifting lateral side). | 1 set of 10 repetitions maintaining each position for 1 second. | 1: Sitting  1x10 repetitions;  2: Standing  1x10 repetitions  3: Standing  1x20 repetitions | Being able to perform the set without pain or muscle cramp after the completion of the set and without loss of balance. | 1-2 minutes |
| **Cotton ball grab**  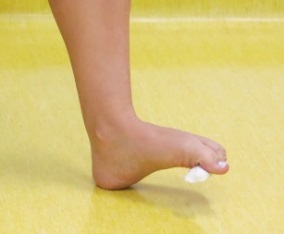  **Rubber ball grab**  **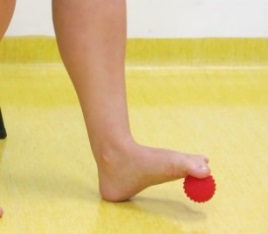**  **Pen grab**  **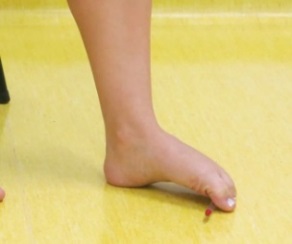** | While sitting, with the heel in a fixed position, grip the object with the toes, lifting off from the ground and placing it back to its original position. Do the same with the other foot. | 1 set of 10 repetitions each foot. | 1: 1x10 repetitions with cotton ball;  2:2x10 repetitions with rubber ball;  3: 3x10 repetitions with a pen. | Being able to perform the set without pain or muscle cramp after the completion of the set | 3-6 minutes |
| **1-5 toe alternate**  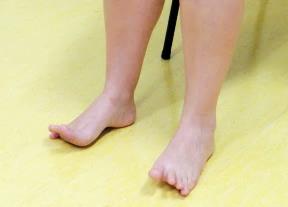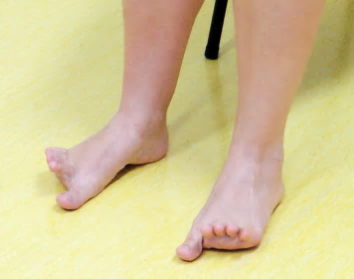 | Sitting, with the heel fixed and contacting the floor, alternately pull the hallux and the little toe on the floor. Do it slowly and under complete control. | 1 set of 10 repetitions each foot, maintaining finger pressure on the ground for 1 second. | 1: Sitting  1x10 repetitions;  2: Standing  1x10 repetitions;  3: Single leg stance  1x10 repetitions. | Being able to perform the set without pain or muscle cramp after the completion of the set and with high control of speed and motion. | 2-3 minutes |
| **Toes abduction**  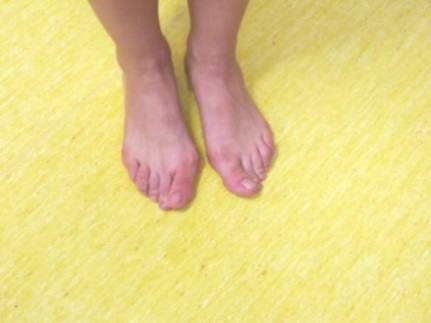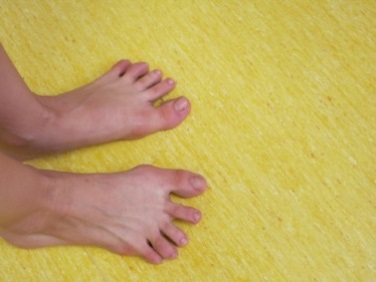 | Sitting, with 90 degrees of knee and ankle flexion, abduct and adduct the toes rhythmically. | 1 set of 10 repetitions each foot, maintaining 2 seconds abducted and 2 seconds on adducted. | 1: Sitting  1x10 repetitions;  2: Standing  2x10 repetitions;  3: Standing  2x10 repetitions maintained for 5 seconds | Being able to perform the set without pain or muscle cramp after the completion of the set and be able to keep the abduction and adduction time. | 1-2 minutes |
| **Plantar arch raise**  **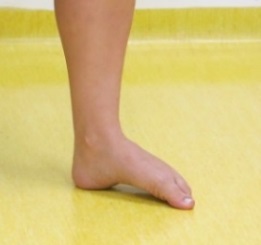** | Sitting, raise the plantar arch in an arch shape. The heel and fingertips should not get off the ground. | 1 set of 10 repetitions each foot, maintaining 5 seconds each contraction. | 1: Sitting  1x10 repetitions;  2: Standing  1x10 repetitions;  3: Single leg stance  1x10 repetitions. | Being able to perform the set without pain or muscle cramp after the completion of the set. | 4-6 minutes |
| **Short-foot exercise**  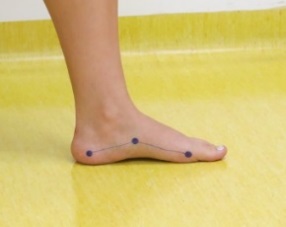  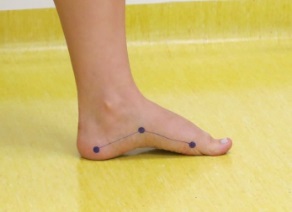 | Sitting, with 90 degrees of knee and ankle flexion, approximate the head of the first metatarsal toward the heel without toe flexion, “shortening” the feet. The forefoot and heel should not get off the ground. | 1 set of 10 repetitions each foot, maintaining 5 seconds each contraction. | 1: Sitting  1x10 repetitions;  2: Standing  1x10 repetitions;  3: Single leg stance  1x10 repetitions. | Being able to perform the set without pain or muscle cramp after the completion of the set. | 4-6 minutes |
| **Toes grasping gait**  **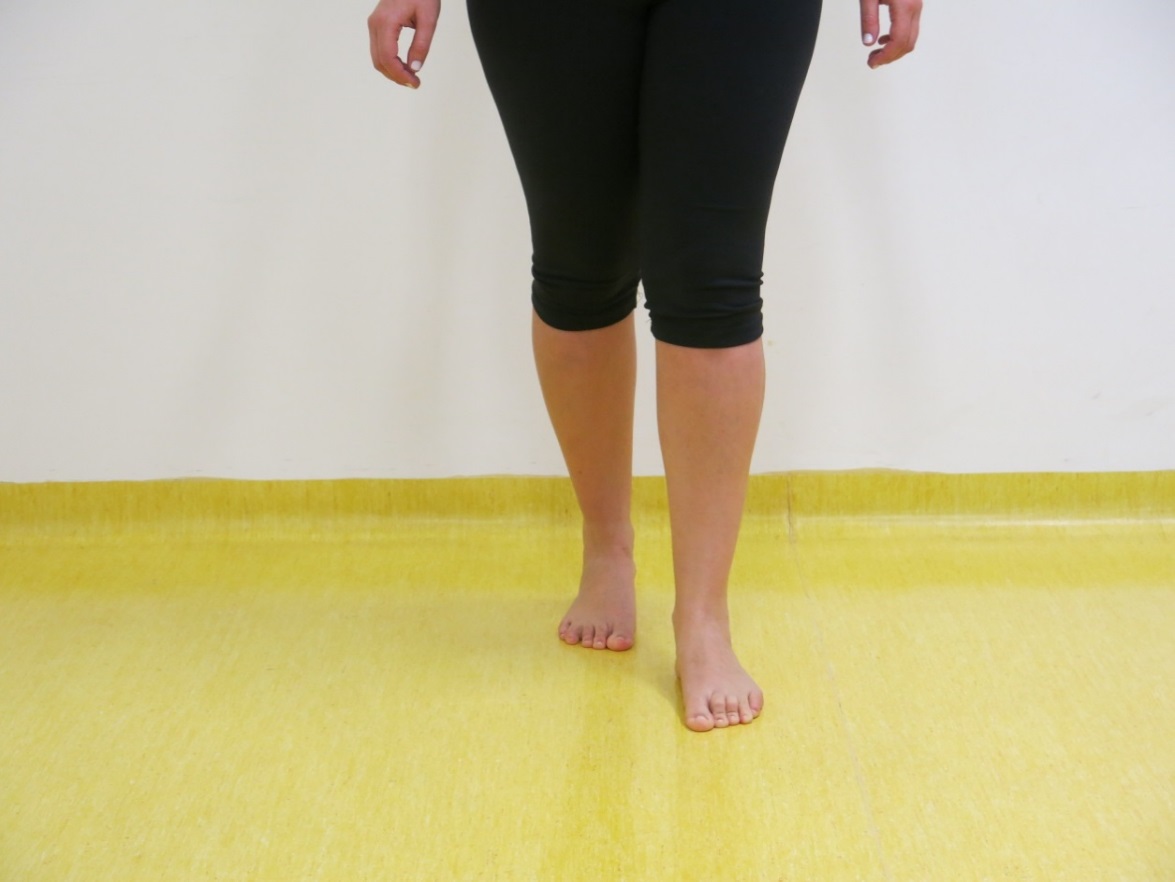** | Walking “grasping” the toes when they touch the ground. Each step grasp for 3 seconds. | 1 set of 10 steps. | 1: 1x10 steps;  2: 2x10 steps;  3: 3x10 steps; | Being able to perform the set in the time described and without pain or muscle cramp after the completion of the set. | 1-3 minutes |
| **Toes abducted gait**  **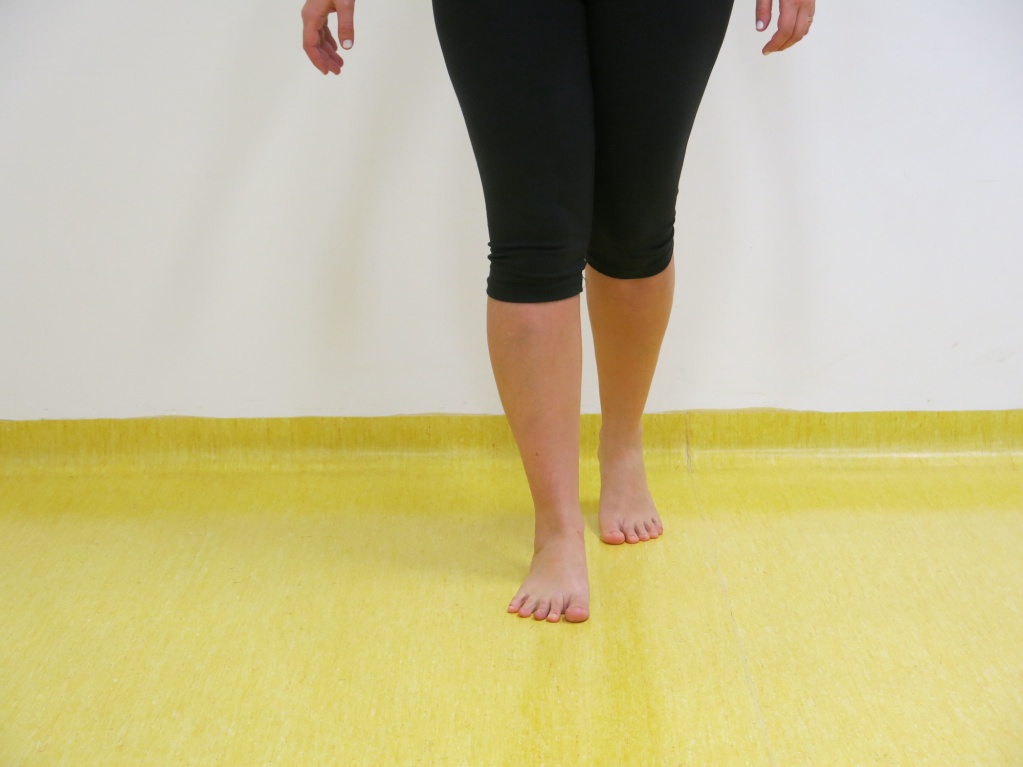** | Walking abducting the toes when the foot touches the ground until take the foot off the ground. | 1 set of 3 steps forward and 3 steps backwards. | 1: 1x10 repetitions;  2: 2x10 repetitions;  3: 3x10 repetitions; | Being able to perform the set without pain or muscle cramp after the completion of the set. | 2-6 minutes |

**Table S3 –** Warm up and stretching exercises.

| **Name** | **Execution** | **Training Volume** | **Approximate Duration** |
| --- | --- | --- | --- |
| **Calf stretch**  **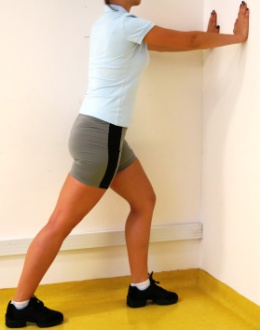** | Standing in front of a wall, keep one leg in front of the other. The front leg with the knee flexed and the rear leg with the knee extended. Lean forward at the ankle, keeping both heel on the ground, stretching the calf muscles. | 1 set of 20 seconds each leg. | 40 seconds |
| **Quadriceps stretch**  **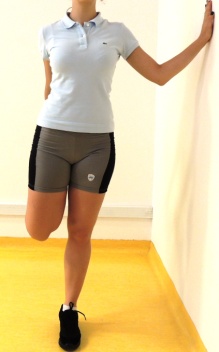** | Standing on one foot, pull the heel towards the bottom, stretching the anterior muscles of the thigh. If necessary, use a wall for support. | 1 set of 20 seconds each leg. | 40 seconds |

| **Fingertip-to-floor**  **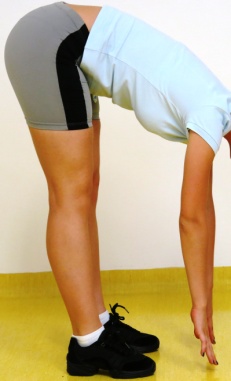** | Standing, with your back straight, bend your trunk forward, keeping the knee straight, trying to touch the fingertip to the ground. | 1 set of 20 seconds each leg. | 40 seconds |
| --- | --- | --- | --- |
| **Lateral stretch (1)**  **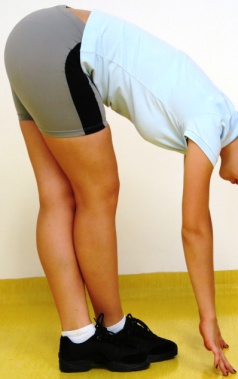** | Standing, with the back straight and with leg crossed over the other, bend the trunk forward, keeping both knees straight, trying to touch the fingertip to the ground. | 1 set of 20 seconds each leg. | 40 seconds |

| **Adductors stretch**  **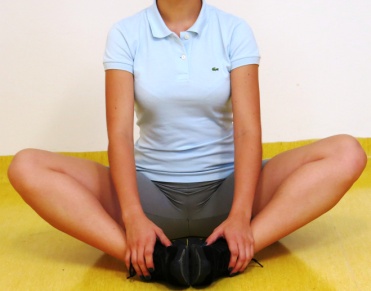** | Sitting, with back straight, knees apart and the sole of feet together, apply gentle pressure to your knees directed to the floor. | 1 set of 20 seconds each leg. | 40 seconds |
| --- | --- | --- | --- |
| **Pretzel Stretch**  **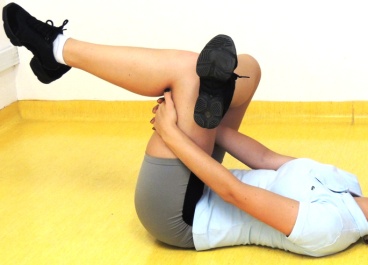** | Lying, with leg crossed over the other, interlace your fingers on the back of the thigh, pulling the leg crossed towards the trunk. | 1 set of 20 seconds each leg. | 40 seconds |
| **Lateral stretch (2)**  **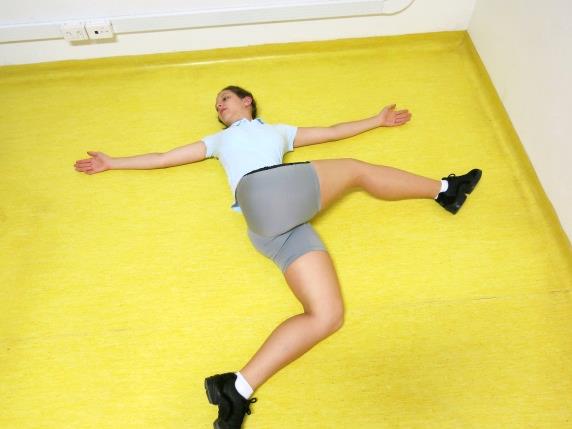** | Lying with open arms, flex and adduct the hip directing the knee to the hand of the opposite side. | 1 set of 20 seconds each leg. | 40 seconds |
